# Salvage Re-transurethral Resection of Prostate as a Diagnostic and Therapeutic Bridge in Treatment-Emergent Neuroendocrine Prostate Cancer: A Multimodal Management Paradigm

**DOI:** 10.7759/cureus.113696

**Published:** 2026-07-30

**Authors:** Lakshmi Kandhan L, Venkata Ramana Korrapati, Roshan Yedulla Reddy, Velmurugan Palaniyandi, Hariharasudhan Sekar, Sriram Krishnamoorthy

**Affiliations:** 1 Urology, Sri Ramachandra Institute of Higher Education and Research, Chennai, IND

**Keywords:** adenocarcinoma, chemotherapy, hematuria, metastasis, neuroendocrine transformation, prostate

## Abstract

Neuroendocrine differentiation of prostatic adenocarcinoma is a very rare and extremely aggressive variant that usually develops after long-term androgen deprivation therapies. Because of the unique biology and rapid progression, early detection of the variant is essential for prompt treatment. We present the case of a male patient in his early 60s who initially underwent transurethral resection of the prostate and bilateral orchidectomy for high-grade metastatic prostatic adenocarcinoma (Gleason 4 + 4, prostate-specific antigen: 44 ng/mL) and had two years of clinical stability. Imaging revealed prostate-specific membrane antigen-avid pelvic lymph nodes and bony metastases, and he subsequently experienced multiple episodes of urinary retention and hematuria. Repeat transurethral resection of the prostate revealed neuroendocrine transformation due to atypical castrate-resistant progression. Systemic chemotherapy and repeated cystoscopies with fulgurations were used to treat our patient, which improved voiding, stabilized the systemic disease, and resolved hematuria. This case highlights the quick evolution of neuroendocrine carcinoma after hormonal therapy and emphasizes that multimodal, individualized treatment can offer meaningful symptom relief and disease control for the patient despite its poor prognosis.

## Introduction

Prostate cancer is one of the most common cancers in the male population. With the introduction of androgen receptor (AR) pathway inhibitors such as enzalutamide and abiraterone, which have greatly improved outcomes for patients with metastatic prostate cancer, the therapeutic landscape has further expanded. Because conventional hormonal therapies work by suppressing the AR signaling pathways, tumors that become AR-independent no longer respond well to these treatments and typically behave more aggressively, the most prominent of which is neuroendocrine prostate cancer (NEPC) [1,2].

NEPC may present in two distinct settings: as a rare de novo malignancy that accounts for less than 2% of cases, or, more commonly, as a treatment-emergent phenomenon following hormonal therapy [3]. The latter is increasingly observed in patients with high-grade adenocarcinoma exposed to prolonged AR-targeted therapy. Treatment-emergent NEPC (t-NEPC) is characterized by small-cell or large-cell neuroendocrine morphology, disproportionately low or modest prostate-specific antigen (PSA) levels relative to tumor burden, visceral metastases, lytic bone lesions, and an aggressive clinical course. The median survival in affected patients is markedly poor, ranging between 7 and 16 months, despite systemic treatment [4].

The pathogenesis of NEPC reflects lineage plasticity under selective therapeutic pressure, with tumor cells adapting to an AR-independent state, in which the tumor no longer depends primarily on androgen signaling for growth, allowing it to progress despite effective hormonal therapies. This change is facilitated by lineage plasticity, i.e., the ability of prostate cancer cells to alter their biological identity and acquire neuroendocrine characteristics. Molecularly, this transformation is often associated with the loss of tumor suppressors, such as *TP53* and *RB1*, PTEN alterations, and epigenetic reprogramming, all of which contribute to the aggressive clinical behavior [5]. Unlike conventional prostate adenocarcinoma, neuroendocrine tumor cells often produce little PSA despite rapid tumor growth and hence rapid clinical or radiological progression is disproportionate to the serum PSA levels. Repeat biopsies are necessary for diagnosis, and management plans are primarily adapted from small-cell lung carcinoma protocols, especially those involving platinum-based chemotherapy regimens [6].

Early detection of NEPC is essential to directing prompt intervention due to its aggressiveness and diagnostic challenges. Although the prognosis is still uncertain, multimodal therapy that combines systemic chemotherapy with local symptom relief measures can offer significant clinical outcomes [7,8]. Here, we report a rare instance of a 62-year-old man who was diagnosed with high-grade prostate adenocarcinoma and had an early neuroendocrine transformation within three years. His condition improved with prompt multimodal therapy, highlighting the need for careful monitoring, pathological re-evaluation, and customized management strategies in such aggressive variants.

## Case presentation

**Figure 1:**
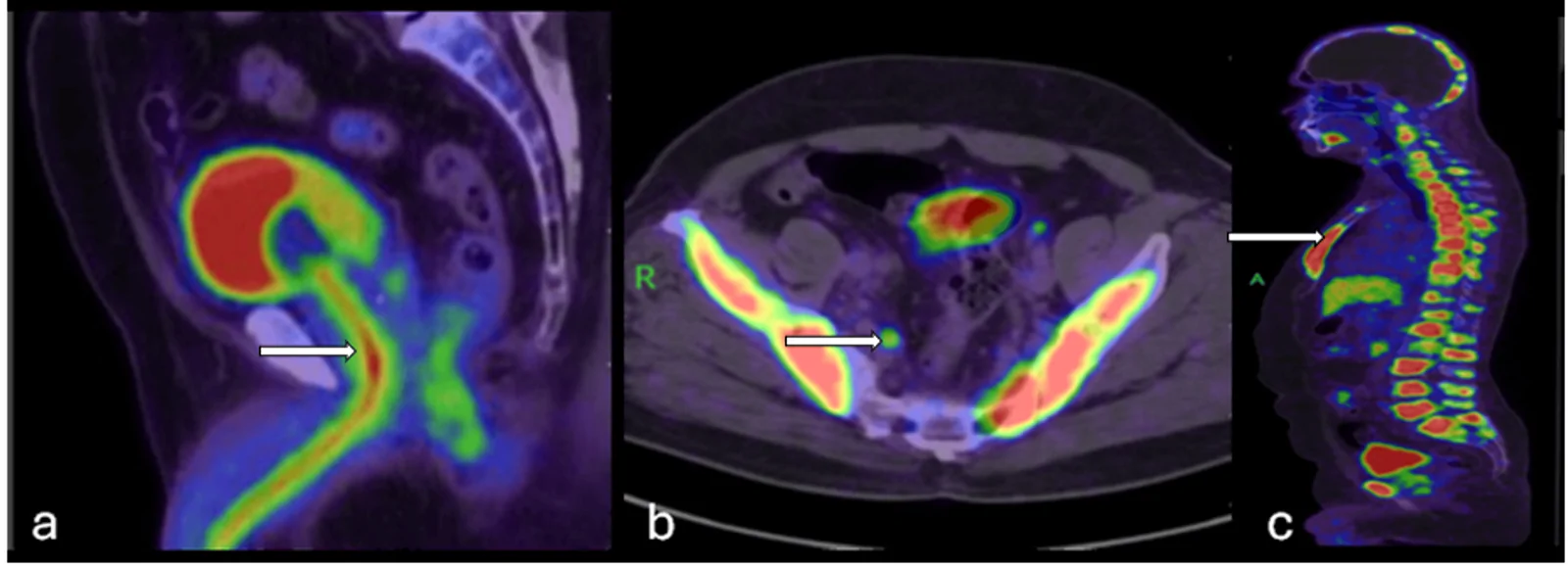
PSMA PET-CT findings during the patient’s clinical course. (a) Baseline sagittal image showing an enlarged prostate measuring 47 × 50 × 21 mm with mild intravesical extension (white arrow). (b) Axial image showing PSMA-avid right internal iliac and left external iliac lymph nodes, with additional non-PSMA-avid right obturator and external iliac nodes (white arrow). (c) Whole-body sagittal image showing multiple PSMA-avid lesions noted in the skull, spine, ribs, pelvis, scapula, clavicle, and sternum, likely metastases (white arrow). PSMA = prostate-specific membrane antigen; PET-CT = positron emission tomography-computed tomography

A 62-year-old man came to us complaining of a weakened urine stream and increased frequency of urination for 30 days. A nodular prostate was found by digital rectal examination. At month zero, a biopsy guided by transrectal ultrasound revealed high-grade prostate adenocarcinoma with a Gleason score of 4+4 (Grade Group 4). At baseline, his serum contained 44 ng/mL of PSA. An enlarged prostate with fluorodeoxyglucose uptake in the left posterolateral aspect and distant bony metastases (cT2aN1M1) was shown on a staging positron emission tomography-computed tomography (PET-CT). The prostate had mild intravesical extension and was asymmetrically enlarged (47 × 50 × 21 mm) (Figure 1, Panel a).

Initial treatment and response

At month zero, the patient was diagnosed with high-grade metastatic prostate adenocarcinoma (Gleason 4+4, Grade Group 4) with nodal and distant skeletal metastases (cT2aN1M1) on staging PET-CT. Given the metastatic stage at diagnosis, the primary oncological intent was systemic disease control using androgen deprivation therapy rather than curative local surgery. Therefore, radical prostatectomy was not pursued, as it is generally reserved for localized disease in appropriately selected patients. The patient was initiated on doublet therapy with abiraterone (androgen biosynthesis inhibitor) and enzalutamide (androgen receptor pathway inhibitor). In addition, the patient was symptomatic with significant lower urinary tract symptoms/bladder outlet obstruction, for which a channel transurethral resection of the prostate (TURP) was performed as a palliative procedure for de-obstruction and symptom relief, and not as definitive cancer surgery. The patient underwent TURP with bilateral orchidectomy. Postoperatively, his serum PSA declined significantly to 0.55 ng/mL, indicating a favorable initial response. He remained clinically stable for approximately 20 months.

Disease progression

At month 21, his PSA increased to 1.6 ng/mL. The patient was on close follow-up, and at 24 months, he developed acute urinary retention. His PSA levels rose to 37 ng/mL. Prostate-specific membrane antigen (PSMA) PET-CT showed an enlarged prostate (49 × 44 × 54 mm) with diffuse PSMA uptake. There were a few PSMA-avid lymph nodes in the right internal and left external iliac regions. Multiple PSMA-avid lesions were seen in the axial and appendicular skeleton, suggestive of extensive metastasis (Figure 1, Panels b, c).

The development of recurrent bladder outlet obstruction and hematuria together with rapidly progressive local disease and clinical deterioration despite prior hormonal response prompted repeat biopsy to evaluate for treatment-emergent neuroendocrine transformation. Although PSA may be an unreliable marker in the setting of neuroendocrine transformation, it did not remain low throughout this patient’s course. Importantly, recurrent urinary obstruction and clinical progression became evident before the later marked increase in PSA to 37 ng/mL. Thus, the disease progression was initially apparent from clinical symptoms and imaging before it was fully reflected by the serum PSA level.

Histopathological transformation

In view of retention of urine and suspicion for neuroendocrine transformation, he underwent repeat TURP (re-TURP). Because our patient presented with acute urinary retention caused by recurrent bladder outlet obstruction, re-TURP was chosen to provide immediate mechanical relief of obstruction while also obtaining an adequate tissue specimen for histopathological re-evaluation. A transrectal or image-guided biopsy in this setting could have established the neuroendocrine transformation, but it would not have relieved the obstruction and might have yielded more limited tissue in a heterogeneous tumor. Thus, re-TURP served both a therapeutic and diagnostic purpose in this symptomatic setting of our patient.

**Figure 2:**
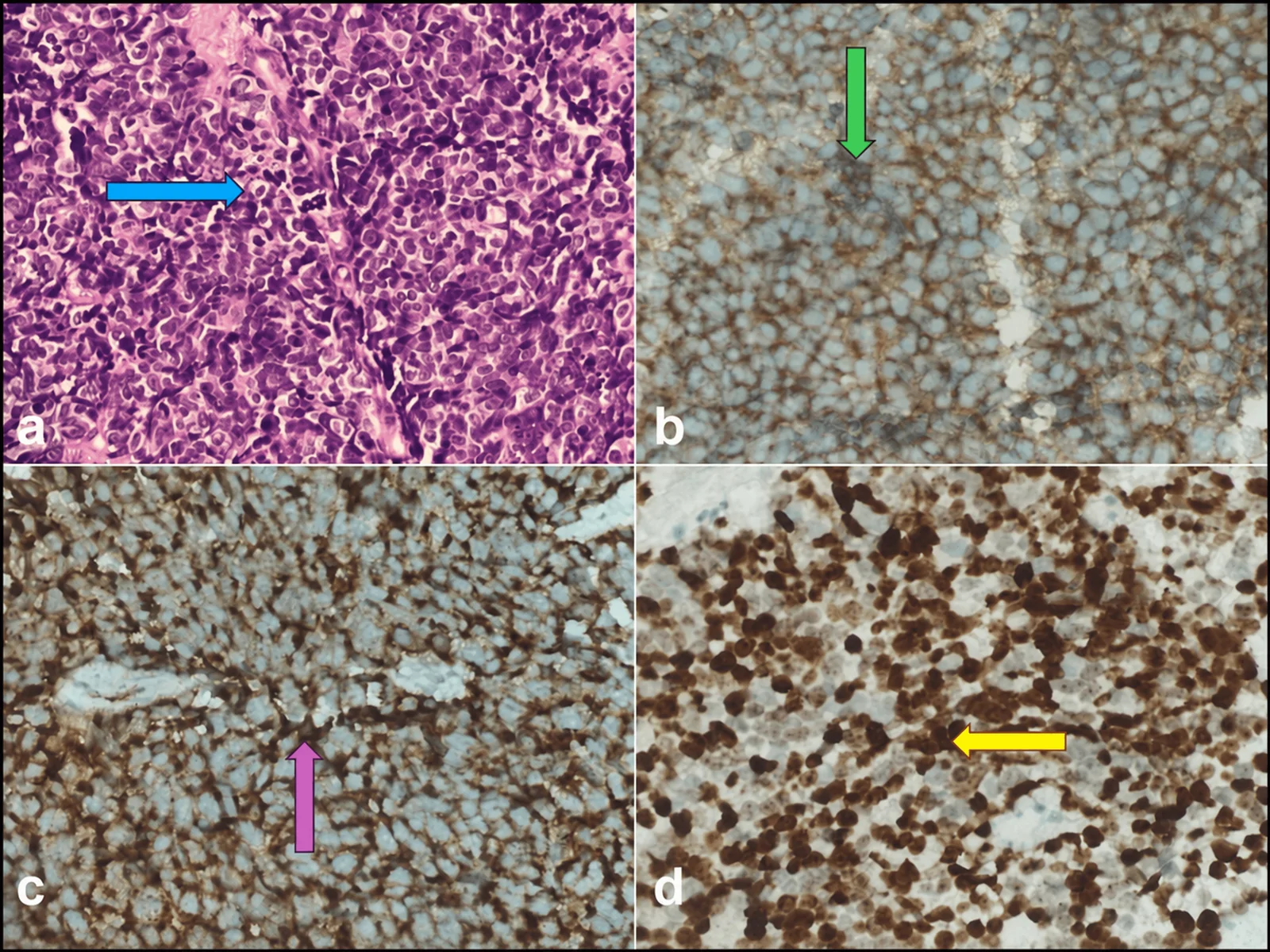
Histopathological and immunohistochemical findings of treatment-emergent neuroendocrine prostate carcinoma. (a) Hematoxylin and eosin-stained section at ×400 magnification showing tumor cells arranged in nests and an insular pattern. The cells demonstrate scant cytoplasm, hyperchromatic nuclei, and marked nuclear atypia, consistent with a high-grade neuroendocrine morphology (blue arrow). (b) Synaptophysin immunohistochemistry showing diffuse cytoplasmic positivity (green arrow). (c) Chromogranin A immunohistochemistry showing diffuse cytoplasmic positivity (pink arrow). (d) Ki-67 immunostaining demonstrating a very high proliferative index of approximately 90%. The findings support neuroendocrine differentiation with highly proliferative tumor biology.

Histopathological evaluation confirmed transformation into prostatic neuroendocrine carcinoma. Immunohistochemistry showed diffuse positivity for synaptophysin and chromogranin A, with a high proliferative index (Ki-67: ~90%) (Figures 2a-2d).

Multimodal therapy and clinical outcome

After histopathological confirmation of t-NEPC, which is typically characterized by reduced dependence on AR signaling, AR pathway inhibitor therapy was discontinued, and systemic treatment was changed to platinum-based chemotherapy with cisplatin and etoposide. The patient received multimodal therapy, including platinum-based systemic chemotherapy, after neuroendocrine transformation was confirmed. Hematuria was managed through local interventions. He received an intravenous dose of cisplatin on day one at a dose of 75 mg/m² and etoposide on days one to three at a dose of 100 mg/m², and repeated the drugs every 21 days in a row, as part of a cisplatin-based regimen. Close monitoring was undertaken for renal function, neuropathy, and ototoxicity, with appropriate hydration and antiemetic prophylaxis. Carboplatin-etoposide was considered a viable alternative in the presence of comorbidities or renal impairment, as both regimens represent accepted standards for t-NEPC.

The bleeding was stopped, urine flow was restored, and follow-up PET-CT performed four months after initiation of chemotherapy showed no new metastatic lesions and no significant increase in the size or extent of the previously identified pelvic nodal and skeletal lesions, and overall functional state and quality of life significantly improved as a result of this integrated approach. In aggressive prostate cancer variants, our case highlights the significance of prompt multimodal intervention in achieving symptomatic relief and disease control. Serum PSA was not monitored after chemotherapy, as PSA is considered an unreliable marker of disease activity in neuroendocrine tumors, and treatment response was assessed mainly through symptom control and follow-up imaging.

The chronology of the patient’s prostate cancer diagnosis, treatment, and follow-up is shown in Table 1.

**Table 1:** Chronological summary of the patient’s clinical course. Timeline of diagnosis, treatment, neuroendocrine transformation, and clinical outcomes. TRUS = transrectal ultrasound; TURP = transurethral resection of the prostate; PSA = prostate-specific antigen; PSMA = prostate-specific membrane antigen; PET-CT = positron emission tomography-computed tomography; FDG = fluorodeoxyglucose; re-TURP = repeat transurethral resection of the prostate

Month	Clinical event
Month (-1)	Patient developed lower urinary tract symptoms
Month 0	TRUS biopsy: high-grade metastatic prostatic adenocarcinoma. Initiation of doublet therapy
Month 0	Channel TURP + bilateral orchidectomy
Month 21	Rising PSA (1.6 ng/mL) on close follow-up
Month 24	Acute urinary retention; PSMA PET-CT: diffuse FDG uptake in the prostate, with skeletal metastasis
Month 24	Re-TURP: neuroendocrine transformation
Month 24+	Initiation of multimodal therapy
Month 24–28	Clinical improvement: resolution of hematuria, improved urinary flow

## Discussion

NEPC displays tumor lineage plasticity and resistance to androgen-targeted therapy. While adenocarcinomas respond well to androgen deprivation therapy and AR pathway-inhibiting agents such as abiraterone or enzalutamide initially, resistant clones can emerge via transdifferentiation into NEPC, an aggressive and androgen receptor-independent variant carrying a poor prognosis [9].

This transformation to NEPC is driven by the combined loss of tumor suppressor genes, such as *TP53* and *RB1*, often coupled with *PTEN* mutations, epigenetic reprogramming and activation of lineage-related transcription factors. Amplification of *MYCN* and *AURKA* has been implicated in facilitating lineage plasticity, enabling tumor cells to bypass AR-driven signalling [10]. In *TP53*- and *RB1*-deficient settings, SOX2 expression drives reprogramming toward a neuroendocrine phenotype, further enhanced by epigenetic regulators such as EZH2 [11].

Clinically, NEPC often presents with aggressive features such as gross hematuria, bladder outlet obstruction, visceral or lytic bone metastases, frequently accompanied by disproportionately low or modest PSA levels. This discordance between PSA and tumor burden is well documented and was evident in our patient, whose PSA rise was modest despite rapid clinical progression. Diagnosis requires a repeat biopsy in cases where disease progression is discordant with PSA kinetics. Small-cell or large-cell morphology, nuclear molds, presence of necrotic areas, higher mitotic activity, and positive results for chromogranin A and synaptophysin markers with high Ki-67 are typical histopathological features [12].

Longitudinally, our patient initially responded to androgen-directed treatment, with PSA decreasing from 44 ng/mL to 0.55 ng/mL and clinical stability for approximately 20 months. At month 21, PSA increased to 1.6 ng/mL, followed by recurrent bladder outlet obstruction and acute urinary retention at month 24, when PSA subsequently rose to 37 ng/mL. Imaging demonstrated persistent locally advanced disease, pelvic nodal involvement, and extensive skeletal metastases. Re-TURP relieved the obstruction and confirmed neuroendocrine transformation, with synaptophysin and chromogranin positivity and a Ki-67 index of approximately 90%. Following platinum-based chemotherapy and endoscopic control of bleeding, hematuria resolved, urinary flow improved, and follow-up imaging showed stable disease without documented radiological progression. However, follow-up after neuroendocrine transformation was limited to approximately four months. At the last documented assessment, hematuria had resolved, catheter-free voiding was maintained, and follow-up imaging demonstrated stable disease. These observations represent the outcome in this individual patient and should be distinguished from the broader literature describing the biological behavior and treatment of NEPC. While the median survival in NEPC is typically only 7-16 months, such multimodal interventions can extend well-being and functional status [13].

Standard chemotherapy protocols include carboplatin plus etoposide in patients with comorbidities, or cisplatin (on day one, 75 mg/m²) plus etoposide (on days one to three, 100 mg/m²) every 21 days. These regimens frequently offer short-term stabilization, but their durability is limited, and toxicity necessitates close observation [14].

This case demonstrates that recurrent endoscopic salvage combined with platinum-based chemotherapy can successfully control severe lower urinary tract symptoms and hematuria following treatment-emergent neuroendocrine transformation of prostatic adenocarcinoma, an approach that has rarely been described in the literature. To our knowledge, this is the first case report to propose salvage re-TURP as a diagnostic and therapeutic bridge facilitating timely multimodal management of t-NEPC.

Emerging therapeutic strategies

Given the poor durability of chemotherapy, novel targeted approaches are being explored. Aurora kinase A inhibitors, such as alisertib, target the MYCN/AURKA pathway; however, phase II studies have shown mixed results, with occasional benefits in molecularly selected patients [15]. In addition, DLL3-directed therapies are showing promise: the bispecific T-cell engager tarlatamab demonstrated an objective response rate of ~22% in DLL3-positive NEPC with encouraging survival outcomes. Epigenetic modulators, such as EZH2 inhibitors, are also being investigated to reverse the lineage reprogramming that underlies AR independence [16].

Comprehensive molecular profiling is increasingly essential for identifying actionable alterations and guiding enrollment into precision-oncology clinical trials, which represent the most promising avenue for improving outcomes in this aggressive disease.

## Conclusions

This case highlights that t-NEPC should be suspected whenever patients develop rapid clinical progression, recurrent urinary obstruction, or hematuria despite previous response to androgen deprivation therapy, irrespective of serum PSA levels. Re-TURP performed for recurrent bladder outlet obstruction can simultaneously relieve symptoms and establish the diagnosis of neuroendocrine transformation of prostatic adenocarcinoma, enabling a fundamental change in systemic therapy. In case of metastatic t-NEPC, multimodal therapy can be effective. This therapy combines platinum-based chemotherapy and repeated endoscopic local control.
